# Progress in Prevention and Treatment of Acute Bone Loss in Orthopedics

**DOI:** 10.1155/2023/9373043

**Published:** 2023-11-30

**Authors:** Haoran Wang, Ji Wu, Boyao Wang, Hu Qin, Lei Fan, Yunhua Wang, Bin He

**Affiliations:** ^1^Department of Orthopaedics, The Second Affiliated Hospital of Nanjing Medical University, Nanjing, China; ^2^Department of Orthopaedics, Sir Run Run Hospital of Nanjing Medical University, Nanjing, China

## Abstract

Orthopedic patients need to perform limb immobilization for several days to several weeks due to fracture or other special circumstances. When the function of a certain part or the whole body is restricted, the activity of osteoclasts will be enhanced and its life activity will surpass that of osteoblasts, so local or even whole body bone loss will occur. Acute bone loss usually occurs within a few weeks after the immobilization of limbs. At this stage, the patient's bone mass will decrease sharply, and the patient is prone to osteoporotic refracture. After that, the bone mass will gradually recover, but the speed of bone formation and bone absorption is difficult to reach a balanced state, and the bone mass of patients will continue to decline after it has recovered to a certain degree. After acute progressive bone loss, a large number of bones were lost and the strength of bones decreased. It is often difficult to recover to the level before fracture for a long time, which undoubtedly increases the risk of osteoporosis and related refractures. According to this common phenomenon of bone loss, clinical treatment varies greatly. After a series of research and practice, clinicians summed up some rules and put forward some feasible suggestions, thus strengthening clinicians' understanding of the treatment of acute bone loss, effectively improving the treatment effect of acute bone loss, having far-reaching significance for preventing and treating osteoporosis, reducing the risk of fracture, and improving the long-term prognosis of patients.

## 1. Introduction

Because the patient's condition needs to be braked for several days or weeks, or even longer, at this time, the activity of a certain part of the body or even the whole body is obviously limited, the body has negative calcium balance, and the activity of osteoclasts is increased, thus covering up the functional activity of osteoblasts, resulting in a sharp decrease of local or whole bone mass. It is a typical acute bone loss phenomenon [1, 2].

In fact, as early as the mid-19th century, some scholars began to pay attention to acute bone loss and Volkmann had observed that patients' bones would undergo certain changes after trauma [3]. At the beginning of the 20th century, Sudeck defined this kind of phenomenon as “acute bone atrophy” for the first time [4]. However, this concept was first put forward by Krølner and Toft in 1983 [5]. At the initial stage of this research, relevant researchers have realized that the bone absorption rate of patients after fracture exceeds the bone formation rate, which leads to bone loss at the fracture site or affected limb. They define this phenomenon as “post-traumatic osteoporosis” or “post-traumatic osteopenia” [6]. In recent years, some scholars believe that the decrease of bone density after fracture is caused by functional apraxia of the affected limb [7]. When the patient recovers from fracture and the affected limb moves normally, the bone mineral density (BMD) can often be rebuilt. However, with the further development of research, scholars have found that fracture not only leads to bone loss in the affected limb but also to bone loss in the contralateral side. This phenomenon often lasts for a long time, and some patients may never recover to the bone mass level before fracture, especially in the elderly [1].

In addition to internal fixation of fractures, other diseases that need to stay in bed for a long time will also have the phenomenon of acute bone loss, which is often an important cause of fractures. Because of this, this phenomenon is highly valued by the majority of orthopedic surgeons and related research is also carried out.

Due to the different cognition of acute bone loss, there are great differences in clinical treatment of this phenomenon, and so far, there is no systematic treatment guide. Therefore, we summarized the views of scholars in related fields on its etiology, pathogenesis, prevention and treatment measures in recent years, and integrated relevant evidence-based medical evidence and clinical experience in order to play a guiding role in the prevention of acute bone loss.

### 1.1. Research Status of Acute Bone Loss

**Figure 1:**
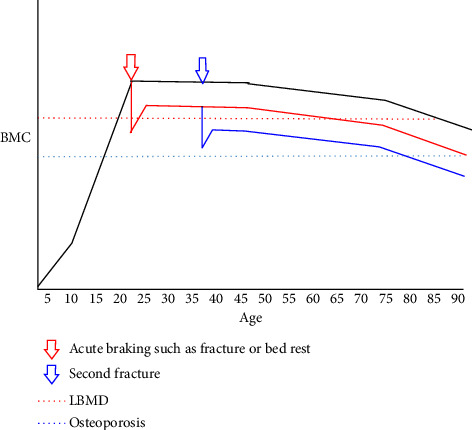
Trends of bone mass with age after fracture: patients have obvious acute bone loss and there will be a long recovery period, but it is difficult to recover to the level before fracture. During this period, patients are prone to secondary fractures and bone loss is further aggravated.

Generally speaking, bone mass is in a positive metabolic stage from childhood to youth and bone mass is in an increasing state. Previous studies have suggested that before the age of 35, the increase of bone mass and bone density will have another plateau and the highest value is called the peak bone mass, which is also the best predictor of osteoporosis in the elderly. However, with the increase of age, the function of bone formation decreases, negative calcium balance appears, and bone mass decreases, which may eventually lead to osteoporosis [1, 8] (Figure 1). Besides age-related bone loss, there are also acute bone loss caused by braking and injury. When patients with low back pain are resting in bed, the weekly bone loss rate is 0.9% of the total bone mass, which is equivalent to the “physiological bone loss” of a normal person for an average of one year after the age of 35, which will increase the incidence of fracture [8]. Relevant experimental research results prove that bone loss occurs immediately after trauma and will continue to increase in the later progress of the disease [9]. It is generally believed that after trauma, the original bone metabolic balance is directly broken, thus the bone density of patients is continuously reduced and the bone microstructure is destroyed. This will not only lead to local or systemic pain but also significantly reduce the bearing capacity of bones. In this case, under the stimulation of mechanical stress, complications related to osteoporotic fractures are easy to occur, which poses a serious threat to the health of patients [10–14]. Therefore, it is of great significance to properly deal with these complications in the treatment of such patients, which has a direct impact on the curative effect.

### 1.2. Common Causes and the Brief Mechanism of Acute Bone Loss

**Figure 2:**
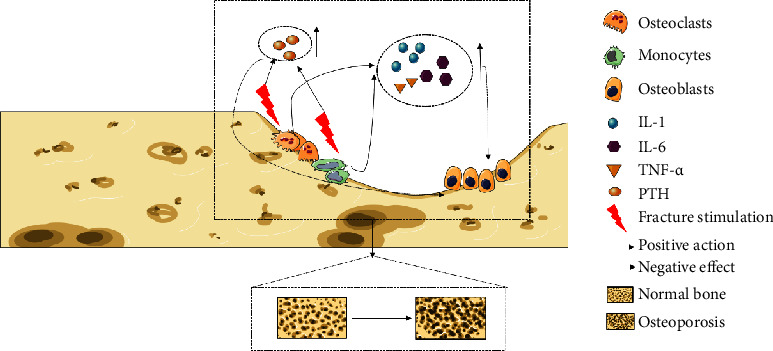
After fracture or fracture, immobilization can easily activate osteoclasts and related immune cells, release a series of cytokines, and inhibit the activity of osteoblasts, accelerating the process of bone loss.

The common causes of acute bone loss are as follows [1, 5, 15–18]: (1) acute braking or plaster fixation caused by fracture and trauma; (2) stress reduction and periprosthetic inflammation caused by joint replacement; (3) spinal cord injury; (4) weightlessness; and (5) conservative treatment of intervertebral disc diseases and long-term bed rest caused by orthopedic traction for various reasons. According to the specific pathogenesis, it is generally believed that this is mainly related to the stress response caused by trauma and some related cytokines. After trauma, osteoclasts are activated under the action of various inflammatory factors and proliferate in large numbers [19, 20], thus cooperating with immune cells to release a large number of proinflammatory factors [21], especially tumor necrosis factor-*α* (TNF-*α*), interleukin-1 (IL-1), and interleukin-6 (IL-6), which have catabolic effects on bone and can inhibit the activity of osteoblasts [22]. Moreover, acute bone loss is not only related to trauma but also to the reduction of stress stimulation caused by limb immobilization (functional disuse), which directly leads to more parathyroid hormone (PTH) secreted by osteoclasts [23]. PTH may be a key regulator of systemic bone resorption, so it is also an important cause of osteopenia (Figure 2).

## 2. Assessment of Acute Bone Loss

### 2.1. Quantitative Evaluation of Acute Bone Loss

During the acute braking period, if the weekly bone loss is ≥0.9% or the total bone loss is ≥3.6%, one of the abovementioned two items can be judged as acute bone loss [5]. For quantitative analysis of acute bone loss, there are mainly the following methods: (1) dual-energy X-ray BMD measurement (DXA) is the gold standard of clinical diagnosis of osteoporosis, and it is also an important content of clinical diagnosis and differential diagnosis of osteoporotic fractures. It can effectively evaluate the state of osteoporotic lesions of fracture patients and has important clinical significance for indicating the risk of postoperative refracture, but it cannot timely evaluate the bone loss of patients in a short time, which is its limitation [24]. (2) Quantitative CT (QCT) is a special method to measure the BMD of patients by using special phantom and software on CT scanner. As for the measurement of BMD, it measures the volume BMD, which is more clinically significant than the BMD measured by DXA. The value of QCT in the diagnosis and treatment of osteoporosis and related fractures has been fully affirmed. It is worth noting that the postoperative curative effect of osteoporotic fractures is closely related to bone structure and bone quality. The preoperative evaluation and postoperative follow-up of QCT can effectively improve the postoperative curative effect of osteoporotic fractures. For example, the absolute value of lumbar QCT bone density >120 mg/cm^3^ is normal, and the value of lumbar QCT bone density ≤80 mg/cm^3^ is a low bone mass [25, 26].

### 2.2. Determination of Biochemical Markers of Bone Metabolism

When acute bone loss leads to osteoporosis and bone lesions, the changes of biochemical markers of bone metabolism are more sensitive and precede the changes of bone mineral density. Biochemical markers of bone metabolism can dynamically evaluate the changes of osteoporosis, which can make up for the deficiency of BMD in the diagnosis and differential diagnosis of osteoporosis. There are many biochemical indices that affect bone metabolism. For patients with acute bone loss after fracture, it is generally recommended to pay attention to the determination of blood calcium, urinary calcium, blood parathyroid hormone, vitamin D, and biochemical markers of bone transformation (serum type I procollagen N-terminal propeptide and serum type I collagen cross-linked C-terminal peptide), among which the determination of urinary calcium is the most commonly used and simplest index [27, 28]. Specifically, patients with fracture or immobilization will have hyperuricemia, which will last for several weeks or even longer and maintained at a high level for a long time. Clinically, it is considered that urinary calcium excretion of more than 4 mg/kg in 24 hours is high urinary calcium [29]. At this time, acute bone loss should be highly suspected.

### 2.3. The Influence of Family History, Disease History, and Drug Application History

Family history, disease history, and drug application history are usually obviously related to acute bone loss and osteoporotic fracture and may even be the direct inducement of fracture [30, 31]. Therefore, the inquiry of patients' family history, disease history (especially fracture history), and drug application history should arouse the attention of clinicians.

## 3. Prevention and Treatment of Acute Bone Loss

Acute bone loss can be divided into osteoporotic bone loss and nonosteoporotic bone loss according to the classification of causes, with the former accounting for the vast majority. Here, we will discuss the treatment measures for the two different types of acute bone loss, respectively.

### 3.1. Treatment of Acute Bone Loss and Pain after Osteoporotic Fractures

#### 3.1.1. Pain Treatment

Acute bone loss can lead to acute pain. Of course, periosteal reaction and acute inflammatory reaction after osteoporotic fracture can also lead to bone pain, so early pain intervention is necessary [32]. The intervention time is generally about 3 weeks. Calcitonin (CT) is a classic choice for targeted treatment, and it can also be supplemented with nonsteroidal anti-inflammatory drugs (NSAIDs) to enhance the analgesic effect [33, 34].

#### 3.1.2. Intervention of Acute Bone Loss

Acute bone loss caused by fracture and immobilization is an important feature of osteoporotic fracture, and it is also a starting factor to further aggravate the malignant cycle of abnormal bone metabolism in osteoporosis. Calcitonin is also the classic drug to intervene acute bone loss in clinic [33]. Calcitonin's main action mechanism is to regulate the activity of osteoclasts and control the secretion of related cytokines so as to effectively slow down the bone loss near fractures and around implants, and at the same time, it can also improve the fine structure of bone and improve the biomechanical properties of bones [35, 36]. Calcitonin can effectively relieve pain, significantly slow down acute bone loss, and also has the effect of nourishing nerves. It was highly praised by doctors in the past clinical application. Now, calcitonin has been withdrawn from the EU market and used as a restrictive condition in the United States (see Table 1 for other related therapeutic drugs).

*(1) Salmon Calcitonin*. Salmon calcitonin can inhibit osteoclast activity and enhance osteoblast activity, thus reducing bone resorption, increasing bone formation, and relieving bone pain. Its specific mechanism of action is as follows: (1) the specific receptor binds to the receptor on osteoclast, which inhibits osteoclast activity, thus inhibiting bone resorption and preventing bone loss. (2) Promote the increase of type I collagen, thus stimulating the proliferation and differentiation of osteoblasts and enhancing the adhesion of osteoblasts. At the same time, it can also prevent the apoptosis of osteoblasts, increase the number of osteoblasts, and promote the remodeling of trabecular bone. (3) It can act on the central nervous system to increase the concentration of *β*-endorphin, and the latter can combine with opioid receptors to produce analgesic effect. (4) Salmon calcitonin can bind to osteoclast receptor and produce a large amount of cyclic adenosine monophosphate (CAMP) in cells, thus activating protein kinase, which can inhibit bone loss and reduce bone pain in patients with osteoporosis. At present, it has been clinically proven that calcitonin can act on the hypothalamus through specific receptors and regulate the pain sensation in the central nervous system. Salmon calcitonin is mainly used in various types of loss and osteoporosis, especially in the treatment of postmenopausal bone loss and painful bone loss. However, salmon calcitonin, as a biological agent, needs to be preserved at low temperature, and at the same time, as a polypeptide drug, it cannot be taken orally and needs intramuscular injection, so allergic reactions may occur, such as facial flushings and adverse reactions of the digestive system or circulatory system. Before using salmon calcitonin, the possible situation and corresponding measures should be explained to patients in order to reduce the influence of adverse reactions and improve the curative effect [35–38].

*(2) Bisphosphonates*. Bisphosphonates (BPS) are antibone resorption drugs, which have obvious effects on inhibiting bone resorption and enhancing bone mass, and have become important drugs for treating bone loss in clinic. With the deepening of research, researchers found that BPS drugs not only play a role in osteoclasts but also play an important role in promoting fracture healing. BPS drugs mainly inhibit bone resorption and reduce bone loss by inhibiting the activity of osteoclasts so as to accelerate callus formation, increase BMD, improve bone turnover rate and calcium balance in the body during fracture healing, and also adjust related biochemical indices.

At present, BPS are generally divided into nitrogen-containing and non-nitrogen-containing types and their molecular mechanisms are also different. Nitrogen-containing diphosphate mainly affects the related enzymes in cells, such as FFP synthase and isoprene (IPP) isomerase, then affects the mevalonate pathway in osteoclasts, and finally affects the bone absorption of osteoclasts. However, the mechanism of non-nitrogen-containing bisphosphonates is mainly that they are metabolized into nonhydrolyzed ATP analogues. These substances can inhibit many intracellular metabolic enzymes and play a role in cell growth inhibition and cytotoxicity. Although their mechanisms are different, both of them can play an antibone loss role against osteoclasts. BPS drugs can be used to treat all kinds of osteoporosis and chronic bone loss; among them, alendronate sodium, risedronate sodium, zoledronic acid, and ibandronate sodium are representative. Alendronate is the most widely used BPS, and ibandronate may also have the effect of relieving bone pain in clinical application. Clinicians usually combine these drugs with salmon calcitonin to enhance the effect [39–42].

*(3) Estrogen/Progesterone*. Changes of hormone levels in early menopause are closely related to accelerated bone loss. From a period of time before menopause, women's BMD will decrease rapidly with age. Studies have shown that the markers of bone turnover in postmenopausal women are higher than those in the control group. Another study pointed out that bone turnover markers are negatively correlated with spinal BMD, while spinal BMD is positively correlated with the serum estradiol level, which indicates that estrogen deficiency may be the key factor of osteoporosis, and if targeted intervention treatment is not carried out, it may lead to postmenopausal osteoporotic fractures [43]. It has been proved that starting estrogen/progesterone therapy as soon as possible after menopause can significantly reduce the incidence of postmenopausal osteoporosis, and long-term application can significantly reduce the incidence of fractures. There are estrogen receptors on osteoclasts and osteoblast cell membranes, but in the process of bone loss, the activity of osteoblasts cannot catch up with osteoclasts, which will lead to greater bone loss, so estrogen mainly plays an antiosteoporosis role against osteoclasts. Estrogen not only stimulates the production of osteoprotegerin (OPG) but also reduces the differentiation of osteoclasts by inhibiting IL-1 and TNF, thus inhibiting the release of M-CSF, RANKL, and IL-6. At the same time, estrogen promotes osteoclast apoptosis through TGF-*β* to achieve the purpose of antiosteoporosis [44]. Previous studies have shown that oral or transdermal absorption of estrogen and progesterone can maintain bone mass and prevent menopause-related bone loss. Comparatively speaking, percutaneous administration can avoid the side effects caused by the first-pass effect of the liver, which can be preferred for patients with obvious cardiovascular risk factors. In a word, the intervention of estrogen combined with progesterone has obvious effect on preventing bone loss and the hormone dose can be adjusted appropriately for different patients. Estrogen therapy needs progesterone to reduce the risk of endometrial hyperplasia; otherwise, patients are prone to irregular bleeding and breast cancer. Although SERM itself may induce osteoporosis, the combination of conjugated equine estrogen and the third generation SERM bazedoxifene has been proved to prevent postmenopausal osteoporosis. Because SERMs can be used as an antagonist of endometrial tissue, estrogen plus bazedoxifene can avoid the related side effects. Therefore, estrogen/progesterone supplementation therapy is mainly aimed at postmenopausal and postmenopausal osteoporosis in women [45].

*(4) Selective Estrogen Receptor Modulator*. Raloxifene, as a classical selective estrogen receptor modulator, has dual functions of estrogen agonist and antagonist. Raloxifene can selectively act on the estrogen pathway, which has a similar effect to estrogen in bone but has no effect on the uterus and breast tissue. Like estrogen/progesterone supplementation therapy, raloxifene is mainly used to treat postmenopausal bone loss or osteoporosis, and its mechanism is as follows: (1) it inhibits the combination of 17*β*-estradiol and estrogen receptors ER*α* and ER*β* and has estrogen agonist activity on bones. (2) It may inhibit the growth of osteoclasts and induce their apoptosis. (3) Combining with estrogen receptor, it can promote the gene expression of the transforming growth factor-*β*3, thus inhibiting the differentiation of osteoclasts. (4) It can increase the reactivity of osteoclasts to calcitonin. (5) Inhibit the production of osteoclast activating factor IL-6. (6) Stimulate osteoblast ossification. Raloxifene can improve the BMD of spine and hip, so it can significantly reduce the fracture risk of postmenopausal women with osteoporosis. Bazedoxifene is a new generation of SERM badoxifene. Its mechanism of action is similar to that of raloxifene, and it competitively inhibits the binding of 17 *β*-estradiol to estrogen receptors ER*α* and Er*β*, and clinical studies have confirmed that bazedoxifene combined with estrogen can prevent bone loss without stimulating the breast and uterus. Lasoxifene may be the most powerful estrogen receptor modulator discovered so far. Studies have shown that lasofoxifene can improve the BMD of the spine of postmenopausal women more effectively, and long-term clinical research data also confirmed that lasofoxifene has a continuous improvement effect on BMD. Compared with raloxifene, it can significantly improve bone resorption and lumbar BMD, so lasofoxifene is probably another potential choice for the treatment of osteoporosis [46–48].

*(5) Parathyroid Hormone Analogue Teriparatide*. Teripartite is a parathyroid hormone-related peptide analogue, that is, recombinant human parathyroid hormone 1–34, which is composed of the first 34 amino acid fragments of parathyroid hormone molecule, and it is also one of the few drugs for promoting bone formation in the treatment of osteoporosis at present. As an agonist of parathyroid hormone PTH1 receptor, teripartite mainly induces the proliferation and differentiation of osteoblasts through two signal transduction pathways, i.e., camp-dependent PKA and calcium-dependent PKC. The molecular mechanism of the action of teriparatide on bone is not fully understood, which may be related to the conformational selectivity of the PTH1 receptor to its ligand. The PTH1 receptor has two different high affinity conformations, namely, the longer signal response selectively binds to the RO conformation and the shorter signal response selectively binds to the RG conformation. Abaloparatide is also a recombinant human parathyroid hormone 1–34. Its function is similar to that of teriparatide, and its affinity for RG conformation is roughly equal to that of teriparatide, but its affinity for RO conformation is much worse than that of teriparatide, so abaloparatide is likely to receive the same clinical treatment effect as teriparatide. Teripartite is a new antiosteoporosis drug, which can induce osteoblast proliferation and differentiation, promote bone formation, and increase bone mass. Clinical trials have confirmed that teriparatide can obviously improve the BMD of patients with osteoporosis and reduce the risk of refracture, and it has been included in the guidelines for the diagnosis and treatment of primary osteoporosis. With the deepening of research, abaloparatide with the same mechanism of action will surely become a new choice for osteoporosis patients [49–51].

*(6) Romosozumab*. Romosozumab is a new antiosteoporosis drug which can be used as one of the treatment options for postmenopausal osteoporosis patients. Sclerotin is an inhibitor of bone formation, which blocks the *β*-catenin signal pathway by combining with the components of low density lipoprotein receptor-related protein (LRP), thus inhibiting bone formation. The mechanism of romosozumab is to block sclerotin, so the clinical application of romosozumab provides a brand-new treatment option for osteoporosis for clinicians. In clinical research, related scholars have confirmed the effectiveness and safety of antiosteoporosis and prevention of bone loss. Romosozumab can not only reduce the fracture of the vertebral body, hip, and nonvertebral body for menopausal women with a high fracture risk but also help patients maintain a stable bone density after antibone absorption drug treatment, further reducing the risk of fracture. Therefore, romosozumab treatment is a new treatment to prevent bone loss or osteoporosis. However, there is a possibility of increasing the risk of cardiovascular diseases in ARCH research, so it needs to be used cautiously in patients with cardiovascular diseases. Some scholars suggest that in ARCH research, pretreatment with alendronate before treatment with romosozumab can reduce the occurrence of cardiovascular events; hence, the treatment with romosozumab combined with BPS may be a new treatment method to prevent bone loss or osteoporosis [52].

#### 3.1.3. Reasonable Treatment of Fracture

For the acute bone loss caused by fracture, timely and reasonable treatment of fracture is the top priority of early intervention of osteoporotic fracture. The treatment of osteoporotic fracture should be individualized, and the treatment plan should be different according to the fracture location, fracture type, bone loss, and systemic condition of the patient. Specifically, the two types of fractures that are most likely to cause acute bone loss are vertebral body fractures and hip fractures. The following is a correlation analysis of the two types of fractures.

*(1) Vertebral Fracture*. Vertebral fracture is the most common osteoporotic fracture [53, 54]. If the pain is severe or the effect of nonsurgical treatment is poor and it is not suitable for patients who stay in bed for a long time, surgery should be performed immediately after surgical contraindications are eliminated. Vertebroplasty is the most commonly used treatment scheme. For patients with nerve compression symptoms and severe kyphosis, open surgery can be considered. The pedicle screw internal fixation system can be placed during the operation to maintain the stability of the spine. If the patient has severe osteoporosis and low bone density, bone cement can be injected locally around the pedicle screws to enhance the therapeutic effect [55].

*(2) Hip Fracture*. Hip fracture is the most harmful type of osteoporotic fracture, and the mortality rate of hip fracture in the elderly is very high, especially in patients with nonsurgical treatment, so the correct intervention for hip fracture is particularly important [56]. Summing up the past clinical experience, we should discuss it from the following three aspects: operation time, prosthesis type, and bone cement filling. Generally, hip fractures should be operated within 24 hours after admission. Previous studies have confirmed that patients who are operated after 6 hours after admission have a higher probability of complications than those who are operated within 6 hours [57]. In terms of prosthesis implantation, there are mainly the following three points: (1) plate fixation: the application of the locking plate can significantly reduce the load on the contact surface between the screw and the bone. Compared with the ordinary steel plate, the stability of the locking steel plate is less affected by bone density, so it can have a better fixation effect on loose bones and reduce postoperative complications [58]. (2) Interlocking intramedullary nail is generally preferred for intramedullary nail, which not only helps patients in moving early after operation but also reduces soft tissue injury and bone loss [59]. (3) The selection of bone cement is very important for patients with severe osteoporosis and low bone density, but it has little significance for patients with normal BMD [60].

In more specific research, after artificial hip replacement for hip fracture, some scholars used dual-energy X-ray absorptiometry to evaluate the changes of BMD around the metal prosthesis after transplantation and found that the bone mass around the prosthesis decreased, especially the loss of proximal femur [61–63]. Most reports confirmed that the changes of BMD around the prosthesis could last for 1–1.5 years after artificial hip replacement, but the decrease was the most obvious in the first 6 months [64–67]. The decrease of the bone mass around the prosthesis can eventually lead to the failure of joint replacement and require another artificial joint revision, which manifestations urgently need to find a good method to intervene the decrease of bone mass around the prosthesis. As for the direct mechanism of bone resorption around the prosthesis after artificial hip replacement, scholars have summarized the following three points [68–71]: (1) the stress shielding effect, that is, after artificial hip replacement, the load-bearing force of the hip joint is redistributed from the proximal end to the distal end, which will lead to the reduction of the stress on the proximal bone, which will inevitably affect the formation and reconstruction of this bone and lead to osteolysis. (2) The implanted prosthesis will increase the pressure on the surrounding trabecular bone, especially some cementless fixed prostheses, which are more likely to produce bone absorption because of their larger volume and higher pressure. (3) Long-term bed rest after operation and immobilization of the affected limb leads to disuse osteoporosis.

In recent years, clinical workers have used bisphosphonates in patients after prosthesis replacement (Table 1), which proves that they can reduce the early bone loss around the prosthesis, which provides an effective way for the prevention and treatment of early bone loss around the prosthesis after hip fracture surgery [39–42, 68].

*(3) Late Management of Fracture*. Postfracture management is the most effective measure to prevent osteoporosis patients from refracture, which has important practical significance and considerable economic impact [72]. The later management can be divided into the following points: (1) fall prevention. It can be said that falling is the main inducement of osteoporosis and fracture again, so as far as osteoporosis and fracture patients are concerned, it is very important to prevent falling at all stages and the living environment is essential whether it is light or the neatness of the ground. At the same time, proper introduction of physical therapy such as ultrasound therapy and electromagnetic wave therapy and active lower limb functional exercise can improve lower limb muscle strength and improve exercise balance, which are all effective means to prevent falling [73]. (2) Basic education. Basic patients' education: to educate patients that acute bone loss is likely to aggravate osteoporosis and to guide them to have a balanced diet and nutrition, prevent falls, get enough sunshine, quit smoking and alcohol, and reasonably avoid drugs that lead to bone loss [74]. (3) Medication. For the rapid bone loss of osteoporosis patients, the adult calcium supplement dose is about 1000 mg/d and it should be taken continuously for a long time. At the same time, vitamin D should be supplemented to promote calcium absorption, and it is suggested that the blood calcium and urine calcium levels of patients should be monitored regularly [75].

### 3.2. Treatment of Nonosteoporotic Acute Bone Loss

The prevention and treatment of acute bone loss in nonosteoporotic patients mainly include maintaining a healthy lifestyle, paying attention to relieving bone pain, early functional rehabilitation, drug intervention, and increasing bone mass. In the treatment of acute bone loss, attention should also be paid to the prevention and treatment of progressive bone loss so as to minimize the difficulty of recovering to the normal peak bone mass and recover to the peak bone mass before injury as much as possible. Among them, lifestyle and early functional rehabilitation are the same as the treatment of acute osteoporotic bone loss. However, in terms of drug intervention, the intake dose should be adjusted. It is generally believed that for the treatment of patients with nonosteoporotic acute bone loss, the optimal daily calcium supplement dose for adults is 800∼1000 mg/d, which lasts for at least half a year. At the same time, vitamin D should be supplemented and the levels of blood calcium and urine calcium should be regularly detected [75].

## 4. Conclusion

Acute bone loss is a common phenomenon in orthopedic patients, with various causes and complicated mechanisms. With the deepening of research, people's cognition about it is constantly updated and the risks of fracture and prosthesis loosening caused by acute bone loss are also highly concerned by orthopedic surgeons. This review is based on published related research and clinical experience and describes the phenomenon of acute bone loss, analyzes its etiology and mechanism, and expounds its evaluation, prevention, and treatment to put forward early intervention to prevent and treat bone loss and maximize the recovery of the peak bone mass in patients so as to provide some clinical reference for the discovery and intervention of acute bone loss in orthopedics.

## Figures and Tables

**Table 1:** Classical common drugs for bone loss.

Classify	Representative medicine	Mechanism of action	Indication
Anticatabolic drugs	Salmon calcitonin [35–38] Alendronate sodium [39] Sodium risedronate [40] Zoledronic acid [41] Ibandronate sodium [42] Estrogen/progesterone complementary therapy [43–45] Raloxifene [46] Bazedoxifene [47] Lasofoxifene [48]	Inhibit the activity, proliferation, and bone absorption of osteoclasts; it can combine with specific receptors, rapidly increase the level of *β*-endorphin, inhibit the synthesis of pain-causing substances such as prostaglandins, and play a quick and powerful analgesic role in many aspects Inhibit the osteoclast activity by affecting the mevalonate pathway or cytotoxicity in osteoclasts, thus inhibiting bone resorption Estrogen and selective estrogen receptor modulator can inhibit bone turnover and prevent bone loss	Various types of bone loss and osteoporosis can also relieve bone pain Various types of osteoporosis and chronic bone loss As above As above As above and has a certain relieving effect on bone pain Mainly aimed at female menopause and postmenopausal osteoporosis Mainly used for postmenopausal osteoporosis of women

Osteoanabolic drugs	Teriparatide [49, 50] Abaloparatide [51]	It can activate the PTH1 receptor of parathyroid hormone and then stimulate the activity of osteoblasts and increase the formation of new bone, but excessive use will easily lead to greater bone absorption than bone formation	It is mainly used for postmenopausal osteoporosis of women and has certain curative effect on other types of osteoporosis

Dual action drug	Romosozumab [52]	Antagonize the negative regulation of sclerostin on bone metabolism and ensure the normal conduction of the *β*-catenin pathway	As above
